# Simple, low-cost fabrication of acrylic based droplet microfluidics and its use to generate DNA-coated particles

**DOI:** 10.1038/s41598-018-27037-5

**Published:** 2018-06-08

**Authors:** Md. Mamunul Islam, Amanda Loewen, Peter B. Allen

**Affiliations:** University of Idaho, Department of Chemistry, 875 Perimeter Dr., Moscow, ID 83844-2343 USA

## Abstract

Hydrogel microparticles were copolymerized with surface-immobilized DNA. Particles derived from a microfluidic device and particles derived from mechanical homogenization were compared. The hypothesis was tested that a controlled droplet generation mechanism would produce more homogeneous particles. Surprisingly, the DNA content of both particle types was similarly inhomogeneous. To make this test possible, a simple, low cost, and rapid method was developed to fabricate a microfluidic chip for droplet generation and in-line polymerization. This method used a low-cost laser cutter ($400) and direct heat bonding (no adhesives or intermediate layers). The flow focusing droplet generator produced droplets and hydrogel particles 10–200 μm in diameter.

## Introduction

We used a low-cost microfluidic technique to test whether mechanical homogenization is responsible for inhomogeneous partitioning of cholesterol-modified DNA into droplets. In our previous work^1^, we generated DNA-coated particles for use as biosensors. The particles can be generated by *in situ* polymerization with DNA localized to the surface by a cholesterol modification. During this work, we noticed that particles were polydisperse in size and inhomogeneous in fluorescent DNA content (even among particles of the same size). We wanted to determine if the droplet generation mechanism was responsible for the inhomogeneity. We hypothesized that droplets would contain homogenous DNA content if dispersion occurred in a single step (instead of multiple rounds of breakup as in mechanical droplet generation). We found that mechanical homogenization and microfluidic droplet generation methods produce significant inhomogeneity in the DNA contents of the droplets. The similarity between the two results suggests that amphiphilic molecules dispersed into droplets may be partitioned unevenly irrespective of the droplet formation mechanism. This fact should be considered in future applications of droplet microfluidics and analytical methods based on water dispersed in oil.

To test this hypothesis, we required a microfluidic chip so that we could observe and control droplet generation and confirm a single step mechanism. However, we lacked the facilities to produce conventional PDMS microfluidic chips. We developed a method for generating microfluidic chips that is simple and inexpensive and may find use elsewhere. Microfluidics is the use of devices that control fluid on the 1–1000 µm scale. Microfluidic techniques are a multidisciplinary field. The advantages of microfluidics include minimizing sample and reagent, producing minimal waste, simple workflows, and enabling the miniaturization of reactions. Droplet microfluidics use various methods to create discrete volumes for analysis^2^. This enables applications in biomedical imaging, drug discovery, biomolecule synthesis, therapeutics, and diagnostics^3^. Droplet-based devices generated multifunctional microparticles^4^, and aided single cell studies^5–7^. There are many more potential future applications of droplet microfluidics^8^. However, the barriers to entry are significant.

The capital requirements to prototype a conventional photolithography-based microfluidic device are high. Photolithography requires an extremely clean environment and a UV exposure system suitable for contact lithography. The photoresists and solvents are also a significant expense. After replication molding of a lithographic master into PDMS, the device is activated with an oxygen plasma cleaner, another high-cost device. With the appropriate equipment, lithography and PDMS can achieve resolution down to ~1 µm for between three and forty times higher capital cost than the procedure described here.

We are presenting a low-cost method to build microfluidic chips with resolution on the order of ~100 µm. Several low-cost approaches have been demonstrated to fabricate devices for droplet microfluidics. These include paperfludic approaches^9–11^, thermoplastic fabrication^12,13^, optical adhesive bonding^14^, glass etching^15,16^, and 3D printing^17–20^. Our method uses PMMA which has advantages over PDMS for generating and maintaining droplets. PDMS is an elastomer widely used in droplet microfluidics^21–26^. However, its surface properties are variable and change in contact with different organic solvents^27^. Furthermore, PDMS is permeable to water^28^. PDMS can also absorb hydrophobic small molecules. Many experiments require time-consuming, expensive surface treatment^29^. PMMA is a thermoplastic material with excellent transparency, biocompatibility, non-porosity, durability and low-cost availability. PMMA has attracted the attention of the microfluidic community^30–34^. However fabrication in PMMA is not as easy as PDMS. It is not elastomeric and not easy to cast. It can be cut with a CO_2_ laser^35^, and several bonding methods have been reported, i.e., adhesive bonding^36^, solvent bonding^37^, and thermal bonding^38,39^. However, adhesives and solvents can occlude the channels and introduce additional (potentially unwanted) materials to the device. High-pressure thermal bonding requires an expensive heated press (e.g., a Carver press costs more than $9,000). Our reliable method for thermal bonding uses low-cost equipment. Our chips had excellent bonding strength, durability, and optical transparency. For a comparison of this method in approximate cost and features, see Table 1.

**Table 1:** Comparison of alternative fabrication methods for droplet microfluidic devices.

Technique	Resolution	Cost/chip	Setup Cost	Transparency	Droplet size
Photolithography^51^	<1 µm	$2	$20,000+	Yes	~1 µm
Low cost PDMS^52^	30 µm	$2	$1500+	Yes	~50 µm
3D printing^53^	200 µm	$2	$3,000	Partial	~100 µm
Injection molding^54^	300 µm	$0.1	$1,000	Yes	~100 µm
Paperfluidics^55^	50 µm	$0.01	$400	Partial	50 µm
Pipette/tubing^56^	n/a	$1	$500	No	75 µm
Previous laser cut^35^	100 µm	$1	$20,000	Yes	n/a
K40 laser (this work)	100 µm	$1	$500	Yes	10 µm

Our PMMA chips made droplets and we observed the droplet formation process directly with an optical microscope. We found that the flow focusing design could produce droplets from 10 to 200 μm in diameter. After prepolymer droplet formation, the chip chemically initiated polymerization. Polymerization of the droplets produced hydrogel microparticles. We linked hydrogel microparticles to DNA by including a polymerizable modification (called an acrydite).

The resulting particles were similarly homogeneous compared to previous methods. DNA coated microparticles were also generated by mechanical homogenization^40^. Mechanical homogenization produces a heterogeneous mixture of bright and dim particles. We hypothesized that this might be due to the droplet formation mechanism. During mechanical homogenization, droplets experience repeated cycles of partitioning of DNA between surface and solution followed by droplet breakup. Surface DNA may not be equally partitioned into daughter droplets. The microfluidic mechanism does not have multiple cycles of partitioning and droplet breakup. Surprisingly, both mechanisms produced inhomogeneous DNA content, suggesting that another mechanism is at work.

This work demonstrates a low-cost approach to probing the particle formation mechanism. We present a low-cost microfluidic fabrication, reliable bonding without a bonding layer or adhesive, microfluidic droplet generation, and *in situ* polymerization to generate particles coated with DNA. The low cost and simple protocol make our process easily adaptable to any lab interested in applying droplet microfluidics. Our technique allowed us to determine that droplet generation creates inhomogeneous composition irrespective of either generation mechanism: homogenization or flow-focusing droplet formation. This fact has implications for any droplet microfluidic application where chemicals may partition to the oil-water interface. Scientists in the field should be aware that the content of their droplets may be inhomogeneous.

## Materials and Methods

### Microfluidic chip fabrication

A 1 mm thin cover layer (Extruded XS Acrylic, Aliexpress) and a 2 mm thick channel layer (Plexiglas®, Tri-State hardware) were etched and cut to form microfluidic chips. Supplementary Fig. SI1 shows the assembly for bonding the microfluidic chip components. LaserDRW software was used to create the designs for all chips. A K40 type laser cutter with a 40-watt CO_2_ laser tube was used to cut the designs into the acrylic. Model F-LM1390, by FLM, China was used for this study; comparable K40 cutters are available from various Chinese manufacturers sold on Amazon and eBay. These laser engraver/cutter devices should be treated with extreme care. Laser safety precautions must be followed including laser safety goggles and good ventilation. The device should be regarded as a laboratory or research grade laser, not a consumer device.

In the design, access holes were cut first; the deepest channels were then etched to a depth of 600 µm, and shallow channels were etched last by increasing the laser head translation rate. After the channels were etched, the chip was cut free from the bulk material. The acrylic plugs left in the access holes were punched out with a blunt syringe needle. All features were cut into the thicker 2 mm acrylic (unless otherwise specified). The 1 mm thick acrylic was then placed over the top to seal the channels. Paper spacer was placed around the perimeter of the press to aid in uniform pressure distribution on the chips placed at the center of a heated platform. A spacer of 32 sheets of paper with an uncompressed thickness of 3 mm was used. The whole assembly was then placed in a heated press (Promo Heat, Tamarac, FL, Model HP230B) at 145 °C for 10 minutes under ~60 N of force from the mass of the press plus the applied force on the handle (measured with Vernier force probe). For a schematic and image of the press and assembly, see Supplemental Information Fig. SI1.

### Characterization of the droplet generator design performance

An optical microscope (Omano, microscopes.com, Roanoke, VA) with a 10× objective lens and Toupview software were used to capture and analyze light microscopy images. It should be noted that the triangular profile of the channels can give the appearance of shadows, charring, or blurring under brightfield microscopy and that careful illumination adjustment is necessary. For clearer wide field images, an Olympus PEN E-PL2 with a Nikon Micro Nikkor lens was used.

The acrylic chips were connected to syringe pumps by stainless steel tubing (stainless steel, 20 gauge). Stainless steel tubing was also cut and glued into the channel inlets. Polyethylene micro medical tubing (95 durometer, Scientific Commodities Inc., Lake Havasu City, AZ) was stretched over the stainless-steel tubing to act as connecting joints. Water droplets were injected dispersed oil medium to form the droplets. 5 mL HSW NORM-JECT (Henke-Sass, Wolf GmbH, Germany) syringes were used for oil injection. A 1 mL SOFT-JECT latex free Luer syringe (Henke-Sass Wolf) was used for aqueous injection.

Flow rates were controlled by motor driven syringe pumps (NE-300, New Era Pump Systems, NY USA and KD Scientific 210, Holliston, MA 01756 USA). The aqueous phase consisted of blue food dye in deionized water, and the oil phases were paraffin oil (Fisher Scientific Company, NJ) with 1% Span 80 (Sigma-Aldrich, St Louis, MO USA) to act as a surfactant. Each chip was placed on the light microscope under a 10× objective lens during operation. An image of the droplets produced was first taken 1.5 minutes after the flow rates were altered. Additional images were taken every subsequent 15 seconds for 75 seconds. The aqueous flow rate was varied between 1 and 10 μL/min and the first oil flow rate was varied between 20 μL/min and 100 μL/min. The flow rate of the second oil phase was set to 30 μL/min and held constant for each trial. This test was repeated for chips that had been cut with wide, standard, and narrower channels.

### Particle generation by microfluidics

A microfluidic droplet generator chip was attached directly to syringes using bent syringe needles. The needles were sealed with glue (All-Temperature Hot Glue Stick, Gorilla glue company, Cincinnati, OH, USA). The aqueous phase (dispersed phase) was composed as follows: 89 mM Tris base (Ampresco, OH); 89 mM Boric Acid (Research Product Intl. Corp, IL USA); 2 mM EDTA (Thermo Fisher Scientific); 5 μM of “Acryd.F.DNA” (5′ Acrydite-ATT ATA GCG GCA CAG AGA C-3′ fluorescein); and 5 μM of complementary, cholesterol-modified DNA (5′-CCG ACC TTA GTC TCT GTG CCG CTA TAA T-3′ Chol.). DNA was purchased from IDT, Coralville, IA USA and was used without further purification. The aqueous phase was annealed in a Thermocycler at 85 °C for 3 min then cooled slowly to room temperature. Acrylamide was added to the aqueous mixture to achieve a final concentration of wt 10% (Research Product Intl. Corp, IL USA). Ammonium persulfate (Bio-Rad Laboratories, Hercules, CA USA) was added to achieve a final concentration of wt 0.8%. The first oil phase (continuous phase) was composed of 1% Span 80 (Sigma-Aldrich, St Louis, MO USA) in Paraffin oil (Fisher Scientific Company, NJ USA). The initiator oil phase consisted of 1% Span 80 in paraffin oil mixed with 0.6% TEMED (Bio-Rad Laboratories, Hercules, CA USA). Flow rates were initially set at 1 μL/min aqueous phase, 100 μL/min of the first oil phase, and 30 μL/min of the initiator oil phase. After the droplets were generated and polymerized into particles, the oil was removed by centrifugation. The collection vial was centrifuged at 16,000 × G for 20 minutes. The supernatant oil was removed by pipette. The vial was then refilled with ethanol (Pharmco-AAPER, Brookfield CT, USA), vortexed, and centrifuged in a low-speed centrifuge for three consecutive washes to remove residual oil. The particles were then air dried for 15 min and re-suspended in Tris-Chloride buffer (100 mM, pH 7.6) containing 0.1% Triton X-100 (KODAK Eastman Chemicals, Rochester NY, USA). Fluorescence micrographs were acquired using an LED fluorescence microscope (LumaScope 620, Etaluma, Carlsbad, CA USA) with a 20× lens.

### Particle generation by homogenization

Polyacrylamide particles were also created using the homogenization method, as described elsewhere^40^. Briefly, the DNA-bearing aqueous phase was prepared as per above. The aqueous phase was placed in a 2 mL centrifuge tube with a ¼ inch ball bearing and 1 mL of paraffin oil containing 1% Span 80. The vial was shaken by hand for 10 seconds to obtain droplets of comparable size to those made by the microfluidics chips. The ball bearing was removed, and 8 μL of TEMED was then added to the suspension and mixed by gentle shaking. The vial was then purged with nitrogen for 30–40 min at room temperature. The resulting particles were washed, dried, and photographed as described above.

## Results and Discussion

### Characterization of laser cutter performance

Our low-cost ($400) laser cutter had several limitations. The CO_2_ laser power was low compared to other laser cutters. The laser head showed irreproducible motion at fast cut speeds. This initially contributed to high rates of failure for small channels. Power settings were re-calibrated weekly to achieve reproducible results. Calibration was performed by a rapid and simple operation requiring only ~5 minutes. A piece of PMMA sheet of 1 mm thickness was paced in the laser cutter. Paper backing was removed from the top side only. A straight line was programmed into the software with a 10 mm/sec translation rate. The analog power knob was increased from zero until the backing paper was visibly ablated by the laser. This was easy to see. Acrylic plastic is removed without any light generated; the backing paper produces a bright yellow flare.

At the calibration power setting when the laser barely penetrates 1 mm of acrylic, about 0.7 watts of power were absorbed by the target (based on the rate of acrylic vaporization and the energy of vaporization)^41^. Originally, this power setting was ~40% of the maximum power setting on the power knob. After 3 months, ~50% of the maximum power setting was required to achieve the same result. We note that this is significantly lower than the advertised maximum 40 watts of the laser.

**Figure 1:**
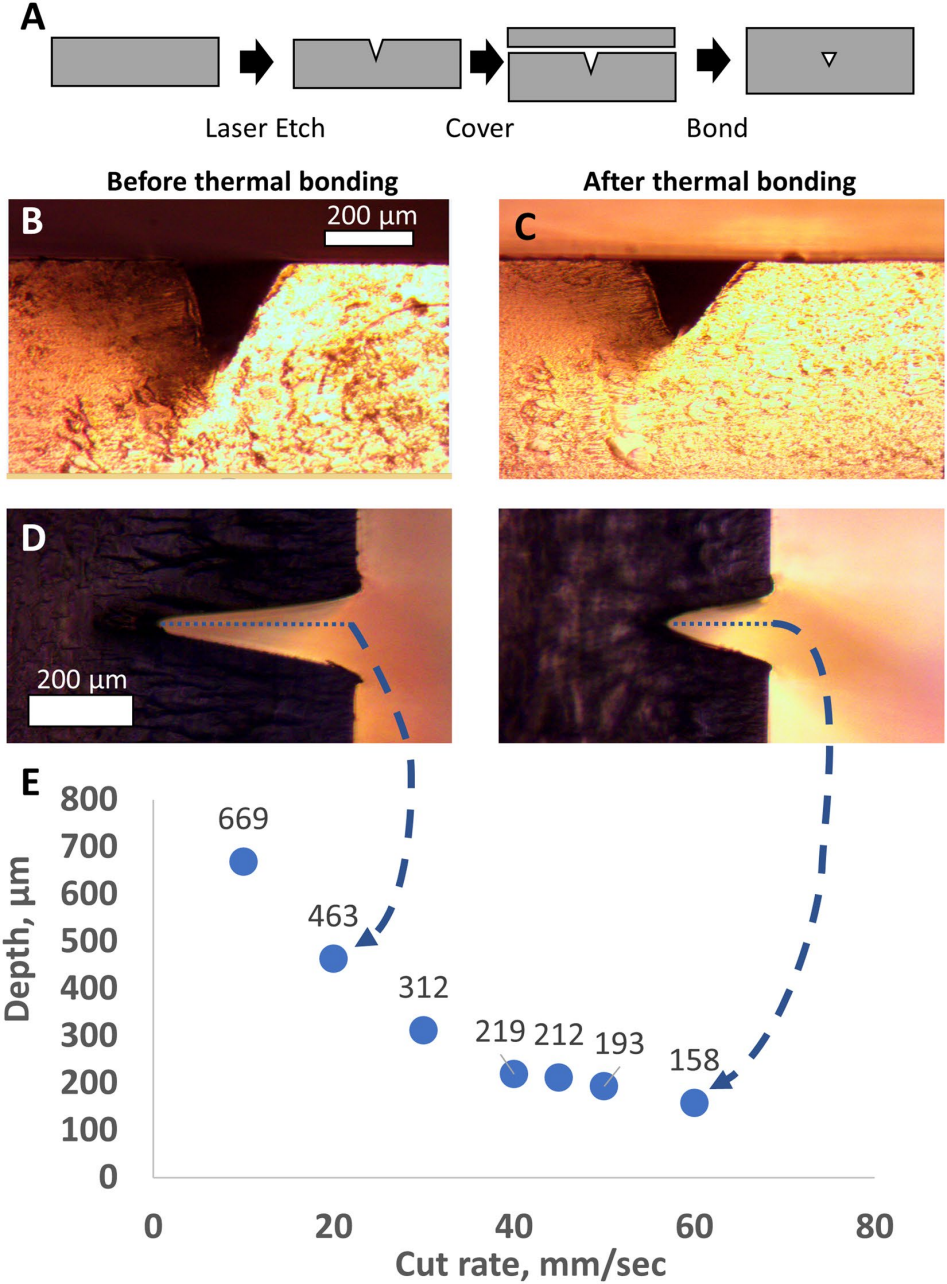
Characterization of laser etching process for generating microfluidic chips. (**A**) Flow diagram of the fabrication and thermal bonding process. (**B**) Cross-sectional images before and (**C**) after thermal bonding. (**D**) Micrographs show the measured depth of the channels in two test cases. (**E**) Graph shows the depth of the fluid channel as a function of laser head translation rate.

When operated with optimal parameters, the K40 laser cutter generated microfluidic devices with reproducible features. We set the laser to the calibrated power setting as described above. At this setting, there was an inverse correlation between the laser head translation speed and the depth of the channels produced (see Fig. 1E). We used this relationship to create chips with desired feature sizes. Before bonding, minimal feature sizes were ~200 μm wide. After bonding, shallow channels were narrower (Fig. 1B,C). We could occasionally produce channels as narrow as 50 μm. Channels ~150 μm wide with 200–300 μm of space between them were produced very reliably (see reliability data in Supplemental Fig. SI3). With careful calibration of temperature and time, smaller channels could be generated as the acrylic fuses and channels shrink under pressure.

### Development of acrylic device bonding protocol

Acrylic chips were etched with a CO_2_ laser and bonded with heat and pressure. Fig. 1A shows a schematic of the laser cutting and thermal bonding processes. Access holes were cut through a 2 mm thick piece of acrylic and successively shallower channels were etched by adjusting laser cut speed. The cut and etched acrylic chip was then covered with a second, 1 mm thick acrylic sheet. The two pieces were bonded with a heated press using heat and pressure as described in Materials and Methods.

We optimized the fabrication protocol by varying the bonding parameters of spacer height, time, and temperature. After numerous iterations of the protocol, we determined the spacer thickness to be the most influential factor of the bonding process. Slight changes (even two sheets of paper or ~90 µm) made the difference between channel collapse and incomplete bonding. With the correct number of paper spacer sheets (32 sheets for the 3 mm thick acrylic assembly), chips could be generated over a range of temperatures (135–155 °C) and times (10–20 min). We found that 145 °C for 10 min yielded the most consistent results as noted in the Materials and Methods. At these temperatures, the acrylic is soft. High pressure flattens the acrylic assembly; the use of the paper spacer maintains assembly integrity.

Images of the channel were captured before and after bonding (Fig. 1D,E). The channel dimensions changed less than 15% with bonding. By adjusting cutting parameters, channels with the appropriate depth can be cut in order to achieve the desired final result. The bonding temperature of 145 °C is well above the glass transition temperature T_g_, 105 °C of the acrylic plastic^42^. At these temperatures, mobile polymer chains diffuse into one another at the interface, forming a strong bond.

We analyzed the applied pressure with Fujifilm prescale tactile pressure indicating sensor film (ultra-low range, 190 to 590 kPa). The film indicated that before bonding, the distribution of pressure was concentrated at the edges. After bonding, we found that the pressure was more evenly distributed across the chip surface (Supplemental Information, Fig. SI2). We interpret these results to indicate that the acrylic assembly deformed and thinned to disperse the applied pressure.

At sub-optimal bonding parameters, the most common failure mode was channel blockage. Failure occurred most frequently within 50 μm of the inlets and at intersections. Channels of width less than 100 μm were associated with mid-channel collapse. Chips etched with large channels often showed a “ridge” of re-deposited acrylic material adjacent to the channel. This ridge was a persistent source of blockage. Blockage frequency was reduced when the deepest channels were etched first and shallower channels etched last. Chips performed best when inlet and outlet holes were cut first, the channel network (deep before shallow) cut second, and the perimeter through-cut performed last. Re-deposition was not a problem for shallow channels. If the etching is carried out in the preferred order, (deep followed by shallow) the small amount of re-deposition from etching shallow channels does not occlude large channels. However, If the device is produced by etching shallow channels first, the large quantity of ablated PMMA produces sufficient re-deposition to occlude the shallow channels. The laser cutter produced features with a minimum width of ~50 μm and a depth of ~100 μm. However, even with optimal bonding settings, small channels showed a high rate of failure. When settings were tuned to produce an etch depth of 200–700 µm, the optimized process showed high reliability (for fabrication success rates at various channel depths, see Supplemental Information, Fig. SI3).

### Particle generator chip behaviour

**Figure 2:**
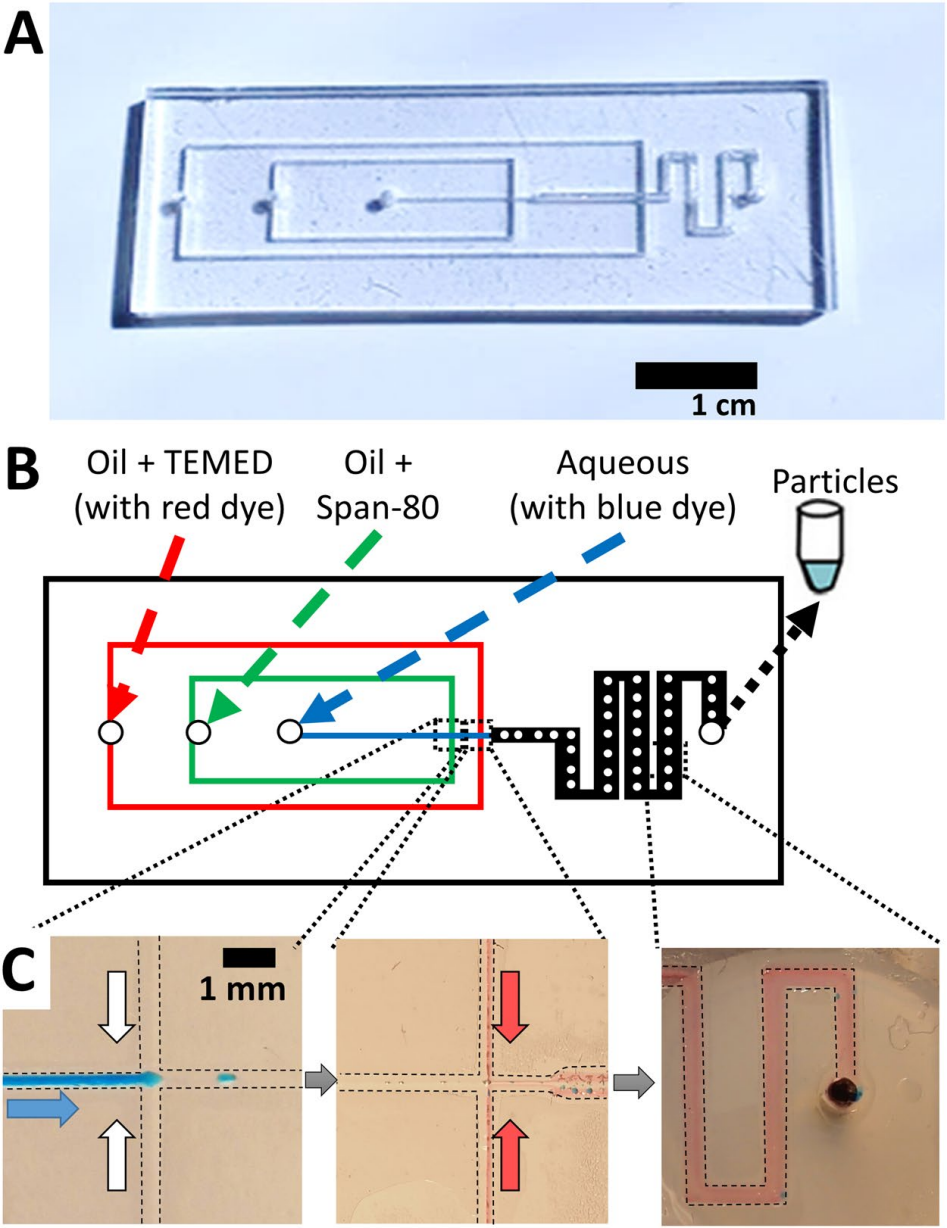
Design and operation of the microfluidic particle generator. (**A**) Photograph shows the finished microfluidic particle generator. (**B**) A schematic shows the design and identity of each fluid inlet and outlet. (**C**) Digital photographs show blue aqueous fluid (indicated by blue arrow) and clear or red oil (indicated by white and red arrows) flowing in the chip. Blue droplets are generated at the first junction. Red oil representing initiator is introduced at the second junction. A mixing region precedes the exit of the microfluidic chip. Dashed lines indicate margins of the channels.

We designed a microfluidic device to generate and polymerize droplets into particles. Our droplet generator used a flow-focusing design. This design introduces a flow of aqueous solution (dispersed phase) into a rapid flow of oil (continuous phase) at a cross junction. The dispersed phase is forced into a narrow cone, which breaks up into droplets. Our droplet generator is shown in Fig. 2A. This design was cut into acrylic sheets and bonded in the manner described above. We connected the device to syringe pumps to deliver oil and aqueous flows as shown in Fig. 2B. For clarity, the aqueous phase was dyed blue and the second oil phase was dyed red. A high-speed movie of droplet generation is also included in Supplemental Information.

Our design introduces oil to the flow at two locations. In the flow-focusing system, the dispersed aqueous phase (with blue dye) and continuous oil phase (clear paraffin oil containing 1% Span 80) were mixed at a cross junction. Aqueous droplets were formed in the continuous oil phase. We left a space of 1 cm between initial droplet formation and introduction of the initiator. This allowed for droplet relaxation to a spherical shape prior to polymerization. Designs produced non-spherical particles when droplet formation and polymerization were too close. Polymerization was initiated by introducing a second oil phase containing TEMED. In Fig. 2C, this second oil phase contained Sudan red to allow confirmation of mixing the oil phases within the device. This design generated water droplets with well mixed red oil at the outlet of the device. We obtained the most homogeneous results when we used blunt syringe needles to directly connect the syringe pumps (see Supplemental Information, Fig. SI4 for a picture of our optimal setup).

### Effect of flow rate on droplet size

**Figure 3:**
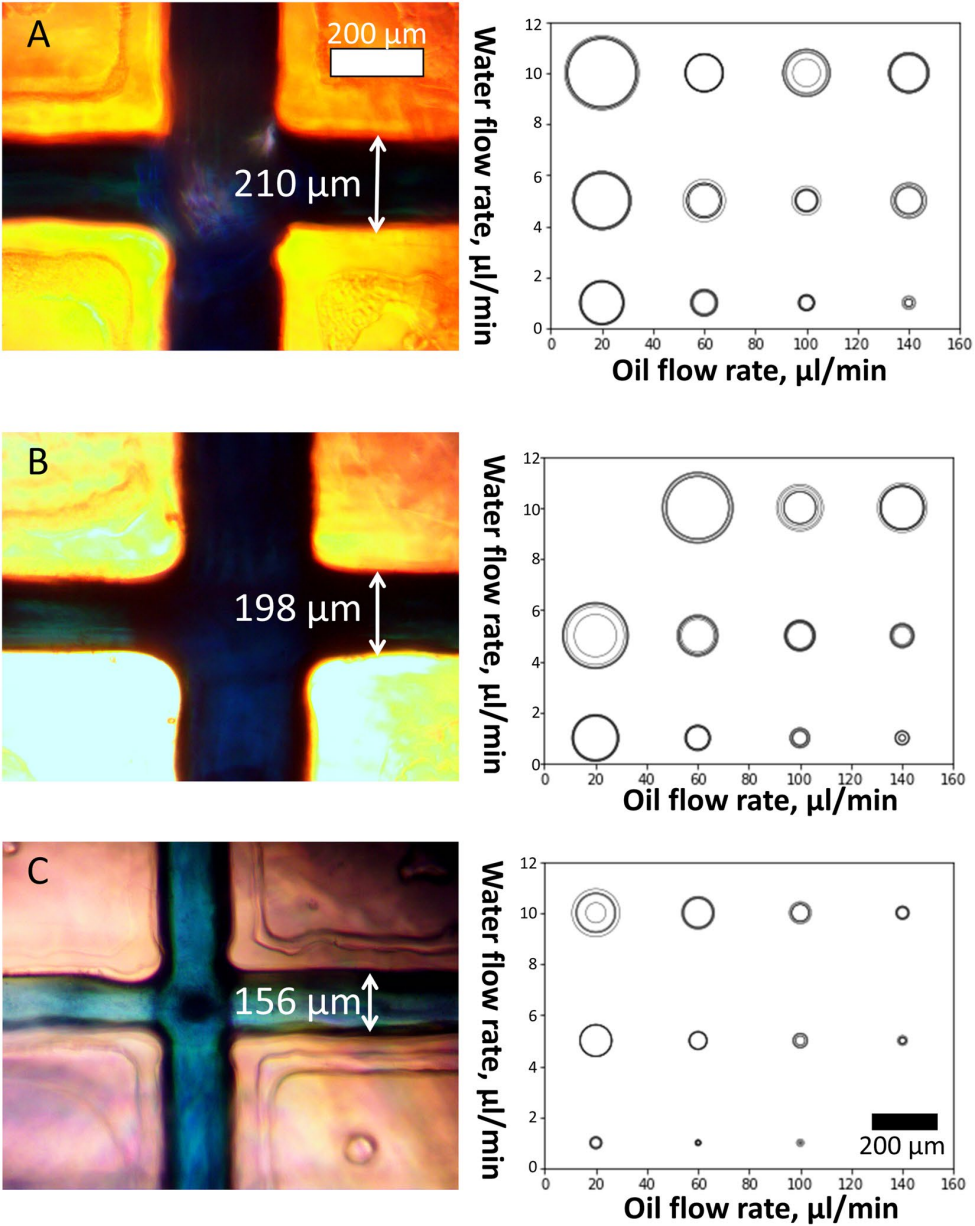
Effect of flow rate and channel size on droplet size. Droplet generator channels are shown at left. Scatter plots at right show the effect of oil and water flow rate on droplet diameter. A 200 µm scale bar is shown in the bottom right. The size of the outlet channel in which droplets were generated was (**A**) 210 μm, (**B**) 298 μm, and (**C**) 156 μm at the base of the triangular channel profile. Flow rate combinations that produced oscillating droplet diameters are represented as concentric circles in the scatter plot, with circle size proportional to the range of droplet sizes produced.

We tested the effects of different flow rates and channel dimensions on droplet size. We generated droplets by using three microfluidic particle generator chips and variable oil and aqueous flow rates. The resulting data are summarized in Fig. 3A–C, shown from largest to smallest (210, 198 and 156 μm wide at the surface, respectively). Some flow rate combinations produced oscillations in the observed droplet diameters.

Flow rate had a greater influence on droplet size than channel size. The size of the droplets ranged from ~10 μm to ~200 μm for all microfluidic particle generators tested. The lower bound for droplet size was defined by the channel dimensions. Smaller channels were required to produce the smallest droplets (<10 μm) at high oil and low aqueous flow rates. Reliable, small droplet generation occurred at an aqueous flow rate of 1 μL/min and an oil flow rate of 100 μL/min. Once a steady state flow rate was achieved, droplet generation became stable. The continuous oil phase flow rate was then increased to 170 μL/min and the oil containing TEMED introduced at 30 μL/min. These parameters were used to generate particles for subsequent experiments.

### Generation of polyacrylamide particles containing DNA

DNA-coated hydrogel microparticles were synthesized by *in situ* polymerization of the droplets generated using the microfluidic particle generator. Acrylamide and Ammonium persulfate were included in the aqueous phase and TEMED in the second oil phase (see Fig. 2).

**Figure 4:**
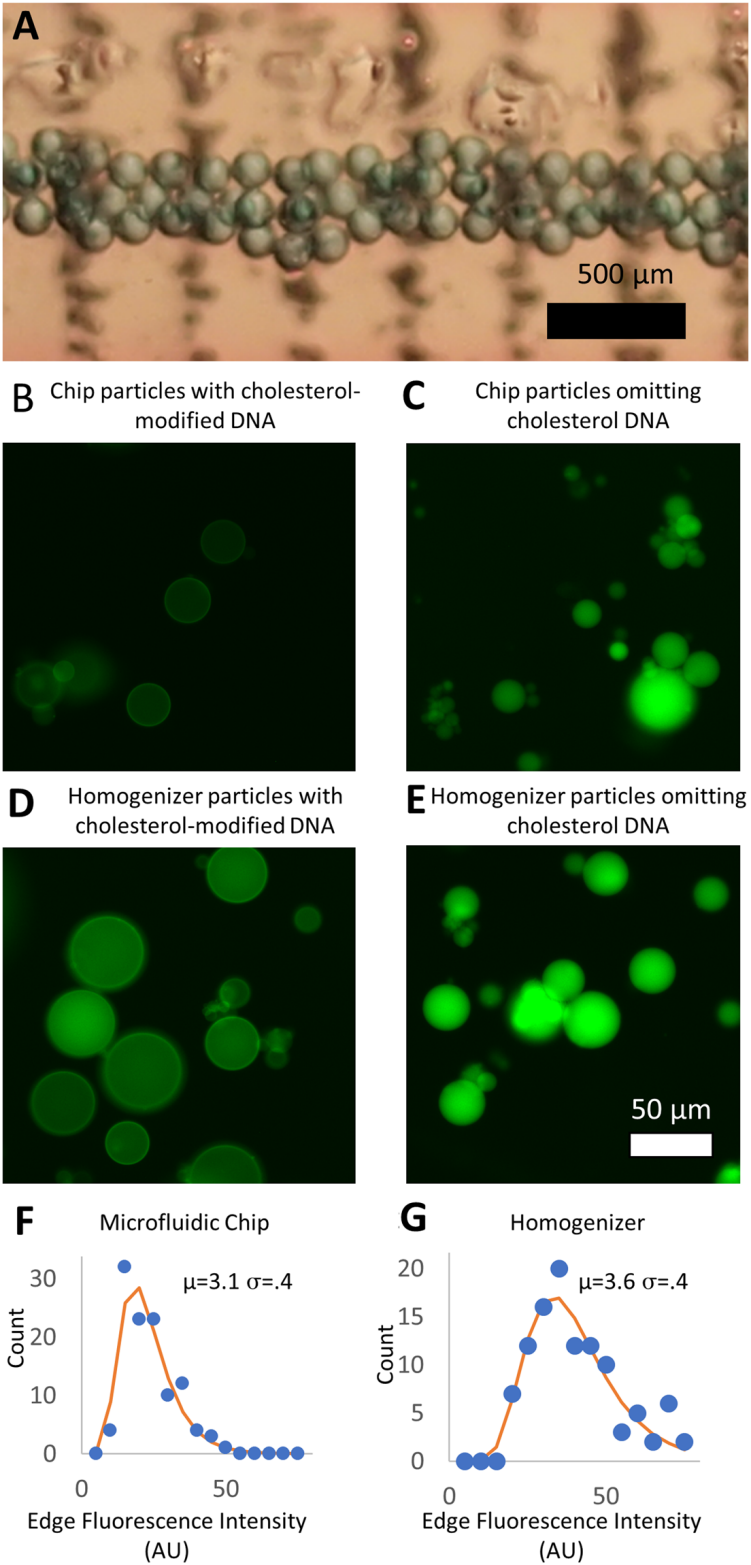
Characteristics of droplets and particles. (**A**) Light microscope image shows consecutive droplets within the particle generator chip connected to syringe pumps. Acrylamide droplets were then polymerized into polyacrylamide particles. (**B**–**E**) Fluorescence micrographs show polyacrylamide particles containing fluorescein-modified DNA. (**B**) Microparticles were generated by a microfluidic chip with cholesterol modified DNA and (**C**) without cholesterol-modified DNA. Polyacrylamide microparticles were also generated using mechanical homogenization (**D**) containing DNA with cholesterol and (**E**) without cholesterol. (**F**) Histograms show the distribution of cholesterol-modified DNA at the surface of particles manufactured using the microfluidic generator and (**G**) those manufactured using mechanical homogenization.

Consecutive droplets are very similar in size. We photographed a set of droplets before they exited the chip and found a standard deviation of their diameters of less than 5% (N = 50, see Fig. 4A). However, when we generated droplets over a span of minutes or hours, the pressure slowly changes (likely due to the pulsation of the syringe pumps). As a consequence, the pooled sample of particles contains a significantly higher polydispersity.

Acrylamide polymerization occurred downstream of the mixing intersection with resultant stable polyacrylamide particle formation. We used an oil flow rate of 150 μL/min, initiator flow of 30 μL/min and an aqueous flow rate of 1 μL/min. The particle sizes ranged from 10–30 μm. Particles obtained from this reaction are shown in Fig. 4B.

The aqueous stream contained DNA modified with fluorescein. The fluorescent DNA confers green fluorescence. The acrydite modification on the same DNA complex has a double bond, which polymerized into the polymer structure. When the DNA is also modified with cholesterol, it localizes the DNA to the surface. The cholesterol prefers the oil phase and so the DNA segregates to the oil-water interface after droplet formation. Polyacrylamide microparticles with cholesterol are shown in Fig. 4B and those without cholesterol are shown in Fig. 4C.

To measure the consistency of the particles’ surface DNA composition, fluorescein-modified DNA at the surface of the particle was imaged using a fluorescence microscope. The fluorescence intensity at the edge of cholesterol-modified particles was quantified with ImageJ. Particles generated by microfluidic chips were similarly consistent in their DNA distribution compared to particles generated by mechanical homogenization. We fit a lognormal distribution to the histogram of particle edge intensities. The sigma (width) term of the lognormal fit was nearly the same for both cases (see Fig. 4F,G).

## Conclusions

DNA-coated particles have been used in diverse experiments, from studies of self assembly^43^ to assays probing DNA methylation^44^ and multiplex PCR suspension arrays^45^. In our previous work, we generated DNA-coated “detector particles” by homogenization^1^. In this work, we have tested the performance of a microfluidic droplet generator for generating fluorescent, DNA-coated hydrogel microparticles using low-cost microfluidics. We polymerized droplets into microparticles coated with DNA within the chip. This microfluidic technique is an alternative method for generating DNA-coated particles for capture and detection of nucleic acid analytes. This method creates droplets through a controlled mechanism that are comparable to mechanically dispersed particles.

We expected to find a difference in the particle DNA content because of the different mechanisms of droplet formation. In the case of microfluidic droplet generation, the composition of the droplet is fixed after generation; there was no subsequent coalescence or breakup. The flow focusing mechanism of droplet formation has been demonstrated in the literature; work with PDMS chips has clearly shown that the flow focusing droplet and particle generation proceeds consistently with our observations^46^. In the case of mechanically homogenized droplets, a given droplet may be broken into smaller droplets multiple times. Every time an unpolymerized droplet breaks apart, there is an opportunity for uneven segregation of DNA to the daughter droplets. However, we discovered no significant difference in the DNA distribution with chip-derived particles. This suggests DNA content inhomogeneity is a result of another, more fundamental process.

Multiple analytical techniques use droplet generation to compartmentalize reactions. Our results suggest that molecules that self-segregate to the oil-water interface may not compartmentalize evenly into droplets. This may have implications for droplet-based technique such as digital PCR^47^ or droplet-based directed evolution experiments^48^.

To test the relationship between droplet formation mechanism and DNA content, we developed a method to create transparent PMMA microfluidic chips with an ultra-low-cost laser cutter and heated press. The device can generate microfluidic chips with features down to ~100 µm wide and ~200 µm deep with a very high success rate. Using a flow-focusing design, these devices could generate droplets and polymerized particles down to ~10 µm in diameter. This technique adds to the repertoire of rapid microfluidic fabrication alternatives such as double-sided tape and paperfluidics. It has the advantage of using only one material (no adhesives or bonding layers). Acrylic devices are well suited to droplet applications. They stretch less than PDMS and have a more stable surface. This low-cost option could help other researchers apply microfluidic approaches.

We can divide the possible mechanisms for the observed inhomogeneity in DNA content into three major categories: fluid dynamics, polymerization, and the chemistry of the oil/water interface. We anticipate that better control over fluid dynamics can produce more monodisperse droplets. A constant pressure source will likely produce better results than the pulsatile pressure delivered by syringe pumps. When consecutive droplets are measured (as in Fig. 4A), they are monodisperse compared to pooled particles from minutes or hours of particle generation (Fig. 4D). Slight variations in pressure over time as the syringe and tubing adjust to the pulsatile pump driven flow may contribute to the observed variability. However, this is unlikely to affect the DNA composition. The dynamics of droplet formation and breakup are vastly different between the homogenized and microfluidically generated droplets. As such, fluid dynamics seem to be an unlikely cause of the common inhomogeneity in surface DNA.

The polymerization and surfactants are the same in both the mechanical and microfluidically generated droplets. The polymerization process was initiated by external TEMED in both methods for particle generation. According to Quong *et al*.^49^, the initiation of gelation from internal chemistry (e.g. with UV photoinitiators as per Yuet *et al*.^50^) can have different effects as compared to external initiation (as in this design). This may be offset by the relatively small particles and short diffusion lengths. The third possibility is that the oil and surfactant chemistry is the cause of the inhomogeneity. In future work, it will be valuable to use mechanical homogenization to explore the possibility of internal initiation of polymerization and a survey of several combinations of oil and surfactant to resolve this question

## Electronic supplementary material

Supplemental video

Supplemental Information
