# Supplementary material for: Simple, low-cost fabrication of acrylic based droplet microfluidics and its use to generate DNA-coated particles
Source: Sci Rep. 2018 Jun 8;8:8763. doi: 10.1038/s41598-018-27037-5 (PMC5993776; doi:10.1038/s41598-018-27037-5)
Supplement: Supplementary file 2 — Supplemental Information [file 41598_2018_27037_MOESM2_ESM.docx]

**Supplemental Information For:**

**Simple, low cost fabrication of acrylic based droplet microfluidics and its use to generate characteristics DNA particles**

**Md. Mamunul Islam, Amanda Loewen, Peter B. Allen*.**

**
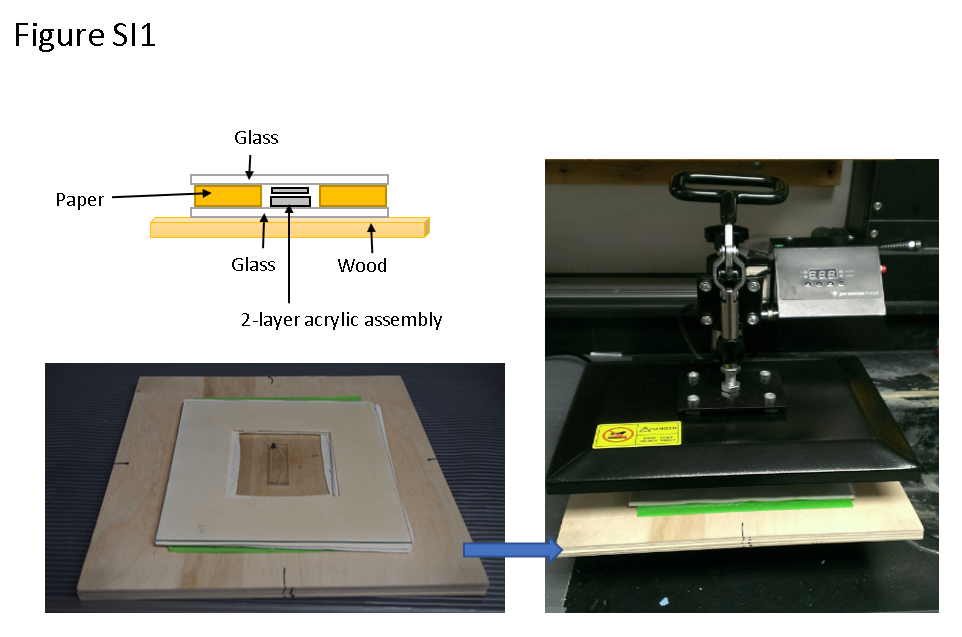
**

**Figure SI1: Heated press assembly. Top let shows a diagram of the assembly used in the heated press. Bottom left shows the assembly before being placed in the heated press. Right shows the assembly after being placed in the heated press.**

**
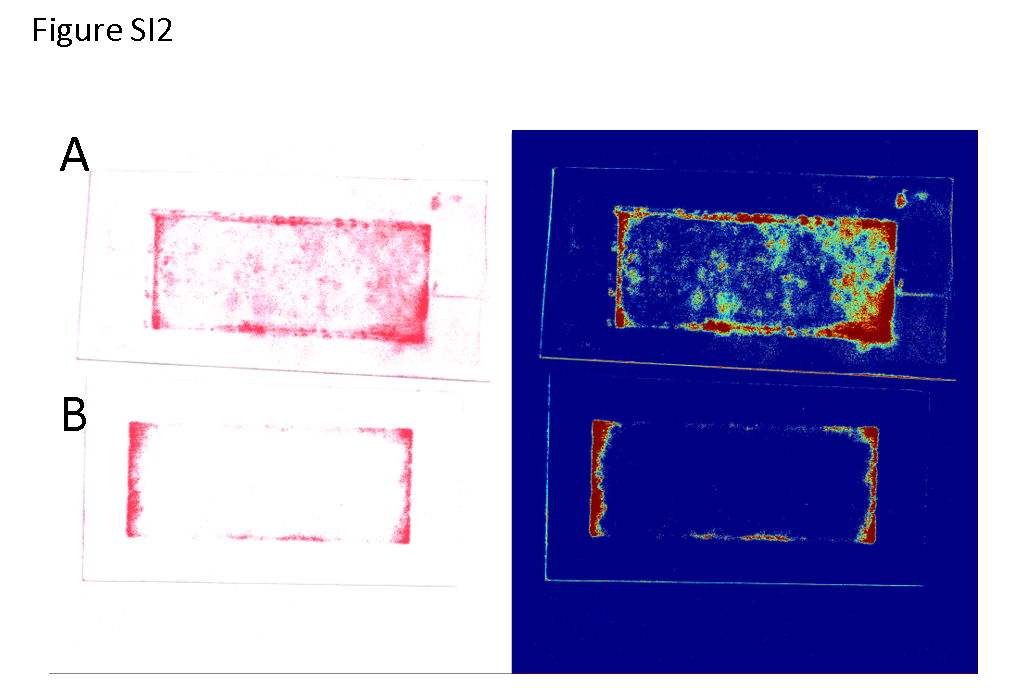
**

**Figure SI2:** Pressure after and before chip sealing. (A) Photograph of pressure sensitive film (28 - 85 PSI dynamic range) placed between the glass and the chip in the heated press after heat bonding the chip and cooling. Left is true color, right is false color to show contrast. (B) Photograph of pressure sensitive film placed between the glass and the chip in the heated press before heat bonding the chip. Left is true color, right is false color to show contrast.

**
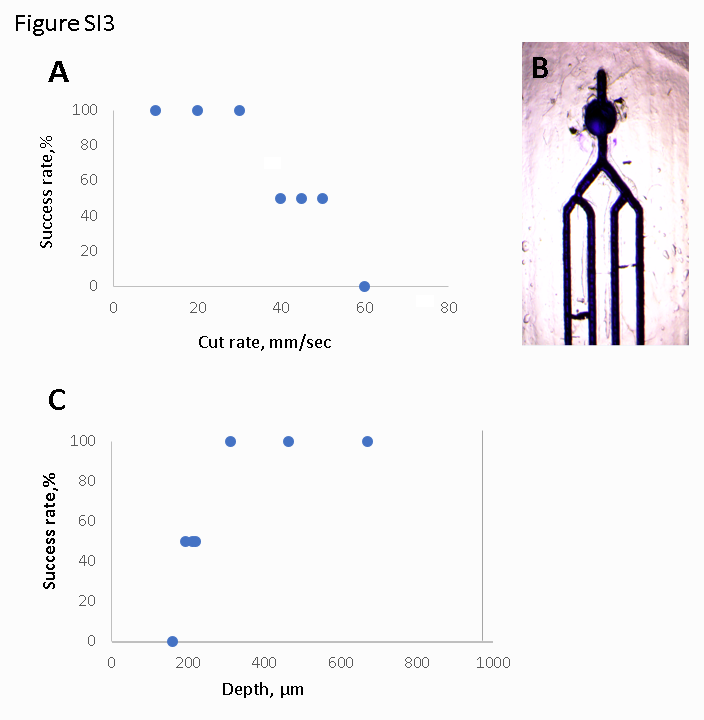
**

**Figure SI3:** Success rate of channels at various conditions. (A) Graph of successful channels as a function cut rate used to fabricate channels. (B) Photograph of the 4-channel chip filled with blue dye. Successful channels filled through to the end; failed channels were blocked. (C) Successful channel % as a function of the channel depth calculated based on the cut rate and calibration curve described above.

**
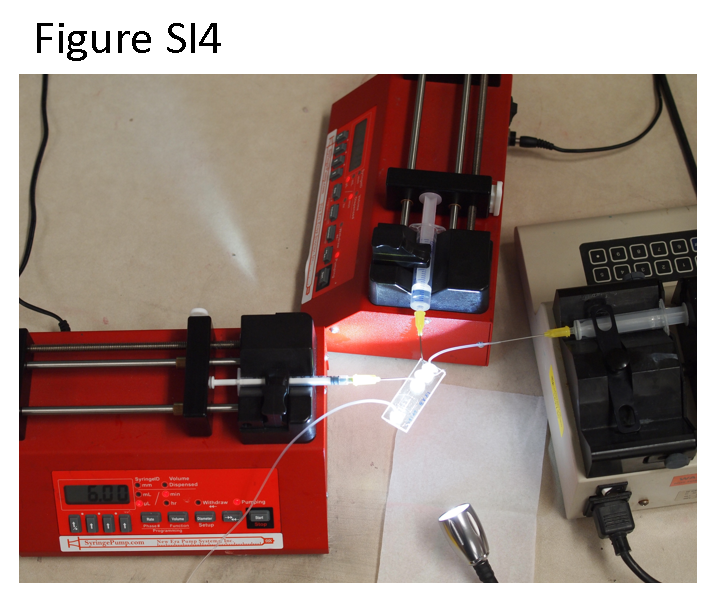
**

**Figure SI4:** Photograph of particle generation chip in operation. Left syringe contains the aqueous phase; top syringe contains the oil phase; right syringe contains the initiator solution.
